# The HIV-1 accessory protein Nef increases surface expression of the checkpoint receptor Tim-3 in infected CD4^+^ T cells

**DOI:** 10.1016/j.jbc.2021.101042

**Published:** 2021-08-04

**Authors:** Rajesh Abraham Jacob, Cassandra R. Edgar, Jérémie Prévost, Steven M. Trothen, Antony Lurie, Mitchell J. Mumby, Alexa Galbraith, Frank Kirchhoff, S.M. Mansour Haeryfar, Andrés Finzi, Jimmy D. Dikeakos

**Affiliations:** 1 Department of Microbiology and Immunology, Schulich School of Medicine and Dentistry, University of Western Ontario, London, Ontario, Canada; 2 Centre de Recherche du CHUM, Montreal, Quebec, Canada; 3 Département de Microbiologie, Infectiologie et Immunologie, Université de Montréal, Montreal, Quebec, Canada; 4 Institute of Molecular Virology, Ulm University Medical Center, UIm, Germany; 5 Department of Microbiology and Immunology, McGill University, Montreal, Quebec, Canada

**Keywords:** HIV-1 Nef, Tim-3, checkpoint receptor, T cell exhaustion, bimolecular fluorescence complementation, ADAMs, disintegrin-like metalloproteases, AP-2, adaptor protein-2, BB-94, Batimastat, BiFC, bimolecular fluorescence complementation, CEACAM-1, carcinoembryonic antigen cell adhesion molecule 1, eGFP, enhanced green fluorescent protein, FBS, fetal bovine serum, Gal-9, galectin-9, HA, hemagglutinin, IFN-γ, interferon-gamma, IgV, variable immunoglobulin, IL-2, interleukin-2, ITIMs, immunoreceptor tyrosine-based inhibitory motifs, LAG-3, lymphocyte activation gene-3, MFI, mean fluorescence intensity, MMP, matrix metalloproteinase, Nef_WT_-V_C_, Venus fluorophore was fused to Nef, PBMCs, peripheral blood mononuclear cells, PD-1, programmed death-1, PFA, paraformaldehyde, PHA, phytohemagglutinin, PtdSer, phosphatidylserine, sTim-3, soluble Tim-3, TCR, T-cell receptor, T/F, transmitted/founder, Tim-3, T-cell immunoglobulin mucin domain-3, Tim-3-V_N_, FLAG-tagged Tim-3, VSV-G, vesicular stomatitis virus G

## Abstract

Prolonged immune activation drives the upregulation of multiple checkpoint receptors on the surface of virus-specific T cells, inducing their exhaustion. Reversing HIV-1-induced T cell exhaustion is imperative for efficient virus clearance; however, viral mediators of checkpoint receptor upregulation remain largely unknown. The enrichment of checkpoint receptors on T cells upon HIV-1 infection severely constrains the generation of an efficient immune response. Herein, we examined the role of HIV-1 Nef in mediating the upregulation of checkpoint receptors on peripheral blood mononuclear cells. We demonstrate that the HIV-1 accessory protein Nef upregulates cell surface levels of the checkpoint receptor T-cell immunoglobulin mucin domain-3 (Tim-3) and that this is dependent on Nef's dileucine motif LL_164/165_. Furthermore, we used a bimolecular fluorescence complementation assay to demonstrate that Nef and Tim-3 form a complex within cells that is abrogated upon mutation of the Nef dileucine motif. We also provide evidence that Nef moderately promotes Tim-3 shedding from the cell surface in a dileucine motif–dependent manner. Treating HIV-1-infected CD4^+^ T cells with a matrix metalloprotease inhibitor enhanced cell surface Tim-3 levels and reduced Tim-3 shedding. Finally, Tim-3-expressing CD4^+^ T cells displayed a higher propensity to release the proinflammatory cytokine interferon-gamma. Collectively, our findings uncover a novel mechanism by which HIV-1 directly increases the levels of a checkpoint receptor on the surface of infected CD4^+^ T cells.

T cells are an essential component of the host immune machinery (1). During viral infection, naïve T cells are activated to create a pool of virus-specific effector T cells (2). Upon cognate antigen recognition, these virus-specific T cells elicit rapid effector functions (2). However, persistent viral replication and antigen stimulation leads to a transient state of T cell dysfunction and exhaustion (3). Although T cell dysfunction is a complex and dynamic process, in general, exhausted T cells lose their effector functions and ability to proliferate and, in severe cases, become prematurely apoptotic (3).

The state of T cell dysfunction was first identified in chronic lymphocytic choriomeningitis virus infection (4, 5). Since then, T cell exhaustion has been characterized extensively in individuals with chronic viral infections including hepatitis C virus and HIV (6, 7, 8). As expected, chronic viral infections jeopardize the host immune response using this immune evasive strategy, among others (9, 10, 11). Indeed, the state of T cell exhaustion positively correlates with disease progression and can compromise viral clearance in antiretroviral therapy–naïve HIV-1-infected individuals (12).

Several checkpoint receptors are expressed on the surface of exhausted T cells during chronic viral infections (3). Collectively, these receptors coordinate the threshold of the immune response by interacting with distinct ligands, thereby regulating T cell signaling. For instance, inhibitory signals are induced in T cells upon the interaction between programmed death-1 (PD-1) receptor and its ligands PD-L1 and PD-L2, promoting functional exhaustion (13, 14). The transmembrane protein receptor lymphocyte activation gene-3 (LAG-3) binds to major histocompatibility complex (MHC) class II molecules and negatively regulates T cell receptor signaling (15). Remarkably, some members of the checkpoint molecule family have canonical immunoreceptor tyrosine-based inhibitory motifs (ITIMs) in their cytoplasmic tail, and phosphorylation of specific tyrosine residues in these ITIMs mediate signal transduction (16). However, other checkpoint receptors are devoid of such residues, rendering their intracellular signaling mechanisms ill defined. Indeed, T-cell immunoglobulin mucin domain-3 (Tim-3) is devoid of ITIMs (17).

Tim-3, a member of the Tim-family proteins, is expressed on activated myeloid cells, including macrophages, and on CD4^+^ and CD8^+^ T cells (18, 19, 20). Structurally, Tim-3 has an N-terminal variable immunoglobulin (IgV) domain followed by a mucin-like domain possessing several O-glycosylation sites, a stalk domain with N-glycosylation sites, a transmembrane domain, and a C-terminal cytoplasmic tail (21). The IgV domain of Tim-3 interacts with multiple ligands using different ligand-binding sites; indeed, at least four different ligands have been discovered for Tim-3: phosphatidylserine (PtdSer), galectin-9 (Gal-9), high-mobility group protein B1, and carcinoembryonic antigen cell adhesion molecule 1 (CEACAM-1) (21, 22, 23, 24, 25). The interaction between Tim-3 and PtdSer enhances the PtdSer-mediated phagocytic engulfment of apoptotic cells (22, 26), whereas Tim-3 interaction with Gal-9 triggers cell death of T helper type 1 immune cells, thereby compromising type 1–mediated immunity (27). Upon high-mobility group protein B1 binding to Tim-3, the innate immune response is suppressed (23, 24). Finally, CEACAM-1 facilitates Tim-3 maturation and surface expression by direct interaction with Tim-3, which is required for the inhibitory function of Tim-3 (25). Interestingly, it has been documented that blocking Tim-3 prevents cancer progression and restores the effector function of T cells during chronic viral infections such as HIV-1, hepatitis C virus, and hepatitis B virus (11, 28, 29, 30).

We and others have determined that Tim-3 decreases HIV-1 infectivity by binding to PtdSer on the surface of HIV-1 virions, preventing viral egress (31, 32). This effect is antagonized by the HIV-1 accessory protein Vpu, which forms a complex with Tim-3 and sequesters it within the *trans*-Golgi network, preventing its plasma membrane localization (32). Because the HIV-1 accessory proteins Nef and Vpu often cooperatively modulate the levels of cell surface proteins such as CD4, CD28, and MHC-I, we sought to determine whether the HIV-1 Nef protein also affects Tim-3 levels in infected cells (33, 34, 35, 36, 37). We determined that the viral protein Nef upregulates Tim-3 on the surface of infected cells, and this is dependent on its highly conserved dileucine motif. We additionally established that Nef and Tim-3 are in close proximity within cells using bimolecular fluorescence complementation (BiFC). Interestingly, shedding of the ectodomain of Tim-3 was moderately enhanced in the presence of Nef, and this process could be blocked by a broad-spectrum matrix metalloproteinase (MMP) inhibitor. Finally, we report that the Tim-3-positive infected population exhibits an activated phenotype, expressing increased proinflammatory cytokine interferon-gamma (IFN-γ). Overall, this study highlights a key role for HIV-1 Nef in dominantly driving Tim-3 upregulation on infected T cells.

## Results

### Nef selectively upregulates cell surface levels of the checkpoint receptor Tim-3

**Figure 1:**
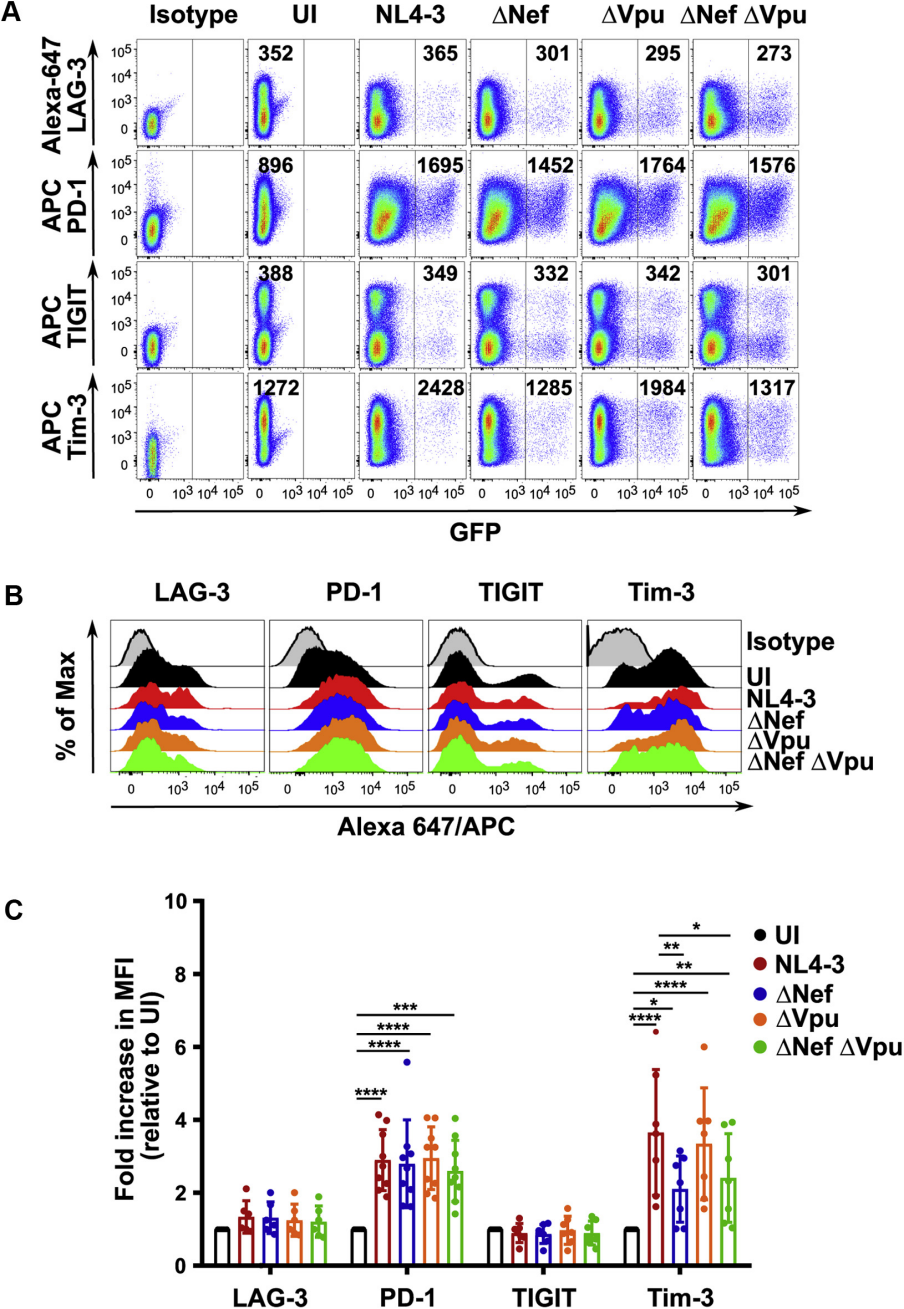
**Flow cytometric analysis of checkpoint receptor expression after HIV-1 infection.** PBMCs isolated from healthy donors were infected with either an eGFP-expressing NL4-3 or a mutant lacking the expression of Nef (ΔNef) or Vpu (ΔVpu) or both (ΔNefΔVpu). Forty-eight hours after infection, cells were surface-stained for the checkpoint receptors LAG-3, PD-1, TIGIT, and Tim-3 using flow cytometry. *A*, representative flow cytometry dot plots illustrating Nef-mediated Tim-3 cell surface upregulation. Infected cells were gated based on eGFP expression, and the mean fluorescence intensity (MFI) for each receptor is indicated within each gate. *B*, representative histograms indicating the expression of each of the four checkpoint receptors in PBMCs infected with the eGFP-expressing NL4-3 or the corresponding Nef and Vpu mutants. For all infected conditions, the eGFP-positive population was used for plotting the histogram. *C*, graph summarizing the fold increase in the expression of the four checkpoint receptors upon infection with NL4-3 or the Nef and Vpu mutant viruses. Values for each receptor are plotted relative to uninfected condition. The values were derived from the MFI of each receptor. ±SD of the mean is indicated (n ≥ 6 experiments from ≥3 donors; ∗*p* < 0.05, ∗∗*p* < 0.01, ∗∗∗*p* < 0.001, and ∗∗∗∗*p* < 0.0001). LAG-3, lymphocyte activation gene-3; PBMCs, peripheral blood mononuclear cells; PD-1, programmed death-1; Tim-3, T-cell immunoglobulin mucin domain-3.

During HIV-1 infection, the surface expression of T cell exhaustion markers positively correlates with viral load and disease progression and was found to be enriched in productively HIV-1-infected cells (12, 19, 38). Thus, the identification of factors modulating their cell surface expression is critical for viral clearance (39, 40). We first investigated if HIV-1 Nef mediates their upregulation. Accordingly, PHA/IL-2-activated PBMCs from healthy donors were infected for 48 h with NL4-3-eGFP or isogenic variants with or without Nef expression and surface levels of four checkpoint receptors: PD-1, TIGIT, LAG-3, and Tim-3, were assessed using flow cytometry (Fig. 1*A*). Overall, the isogenic variants lacking Nef expression had similar levels of virus replication and did not demonstrate any significant reduction in virus replication in PBMCs compared with WT viruses (Fig. 1*A*). For all infections, levels of checkpoint receptors in HIV-1-infected cells (GFP-positive) were compared with uninfected controls (Fig. 1*A*). Infection of PBMCs with NL4-3 WT increased the cell surface expression of the checkpoint receptors PD-1 and Tim-3 about 3- to 4-fold when compared with uninfected controls (Fig. 1, *A*–*C*). In contrast, TIGIT or LAG-3 cell surface levels did not increase upon NL4-3 infection (Fig. 1, *A*–*C*). We next sought to examine if the increases in PD-1 and Tim-3 expression upon infection required Nef (Fig. 1, *A*–*C*). Although PD-1 expression was substantially increased in NL4-3-infected cells, infection with a virus failing to express Nef did not impact this upregulation (Fig. 1, *A*–*C*). In contrast, the upregulation of Tim-3 cell surface expression by NL4-3 was partly Nef-dependent as a twofold decrease in the fluorescence intensity of Tim-3 was observed in cells infected with a virus failing to express Nef (ΔNef) (Fig. 1, *A*–*C*). Because HIV-1 Nef was partly responsible for upregulating surface Tim-3 expression, we further assessed the levels of surface Tim-3 in the infected population (GFP positive) and compared it with the uninfected population (GFP negative) from the same well in cells infected with NL4-3 or a virus failing to express Nef (ΔNef). Importantly, the GFP-positive population expressing Nef had significantly higher levels of surface Tim-3 than the GFP-negative population, suggesting that this effect is due to infection and not due to a bystander effect (Fig. S1*A*). Moreover, as the ability of Vpu from T/F HIV-1 CH58 and CH77 to modulate these receptors has been previously characterized, an HIV-1 NL4-3 virus failing to express Vpu was included (32). In contrast to T/F CH58 and CH77 Vpu proteins, NL4.3 Vpu did not alter Tim-3 cell surface levels, as the fluorescence intensities were equivalent between the WT and the ΔVpu viruses (Fig. 1, *A* and *C*) (32). Critically, both NL4-3 Nef and Vpu were able to downregulate CD4, and Nef was able to downregulate MHC-I efficiently, demonstrating that these accessory genes are functional (Fig. S1*B*).

### Nef upregulates cell surface levels of Tim-3 using its dileucine motif

**Figure 2:**
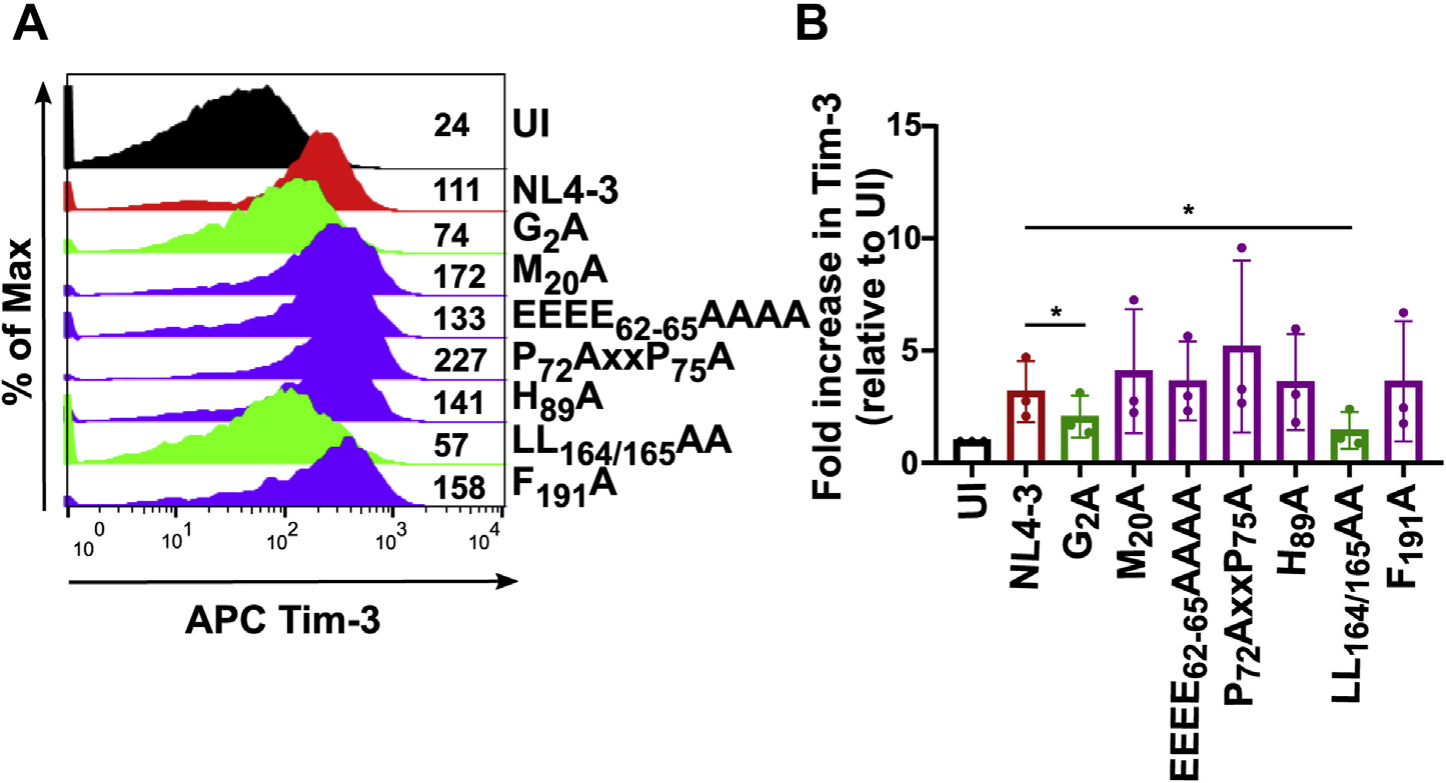
**Nef modulates Tim-3 expression using its dileucine motif.** PBMCs from healthy donors were isolated using negative selection and infected with an eGFP-expressing NL4-3 or various Nef mutants. Forty-eight hours after infection, PBMCs were stained using the Tim-3 antibody and infected cells analyzed using flow cytometry. *A*, representative histogram indicating Tim-3 expression on the cell surface. The eGFP-positive population was used for plotting the histogram. The *numbers* indicate the mean fluorescence intensity of surface Tim-3. *B*, graph summarizing the fold increase in the expression of Tim-3 upon infection with NL4-3 or the Nef mutant viruses. The values were derived from the mean fluorescence intensity of the Tim-3 receptor. ±SD of the mean is indicated. The fold change in Nef-mediated Tim-3 expression relative to uninfected condition is summarized as a graph (n = 3 experiments; ∗*p* < 0.05). PBMCs, peripheral blood mononuclear cells; Tim-3, T-cell immunoglobulin mucin domain-3.

Nef interacts with host cellular proteins using well-characterized motifs to mediate multiple functions including the modulation of host cell surface receptor expression (41, 42). Thus, to determine the precise motifs in Nef responsible for Tim-3 upregulation, we generated a panel of HIV-1 NL4-3 constructs expressing an array of Nef mutants that are impaired in their ability to interact with specific host proteins (41, 42). Based on previously established structure/function studies that mapped interactions between Nef and host cell proteins (41, 42), HIV-1 NL4-3 isogenic viruses encoding seven different Nef variants were used to study Tim-3 upregulation (Fig. 2*A*). Mutation of the N-terminal glycine (Nef G_2_) prevents myristoylation, a function essential for Nef membrane association (43). The methionine residue (Nef M_20_) and the acidic cluster (Nef EEEE_62–65_) motif are essential for MHC-I downregulation by interacting with the vesicular adaptor protein complex 1 (42, 44). The Nef EEEE_62–65_ motif is also involved in binding to the membrane trafficking proteins phosphofurin acidic cluster sorting proteins-1 and -2 (45, 46, 47). Furthermore, the Nef P_72_AxxP_75_A mutant is deficient in binding to members of the Src family kinase proteins and mutation in the H89 (Nef H_89_A) and the F_191_ residues (Nef F_191_A) abrogate Nef’s interaction with the serine/threonine protein kinase PAK-2 (48, 49). Finally, we mutated the highly conserved Nef motif LL_164/165_, a key motif implicated in downregulation of CD4, CD28, and SERINC5 *via* its interaction with the adaptor protein-2 (AP-2) complex (50, 51, 52).

Accordingly, PBMCs were infected with various HIV-1 NL4-3 Nef mutant constructs, and Tim-3 cell surface expression was determined using flow cytometry. Interestingly, mutating the LL_164/165_ motif of Nef abrogated its ability to upregulate cell surface levels of Tim-3 (Fig. 2, *A* and *B*). Unsurprisingly, Nef membrane association was also essential for Nef-mediated Tim-3 upregulation as the virus expressing Nef G_2_A failed to upregulate Tim-3 (Fig. 2, *A* and *B*).

### Nef and Tim-3 are in close proximity within cells

We have previously used a BiFC assay to demonstrate that Tim-3 is in close proximity to Vpu (32). Thus, we evaluated if Nef can form a complex with Tim-3. BiFC takes advantage of the fusion of two proteins of interest separately linked to split fragments of the Venus fluorophore (V_N_ or V_C_) (53). Once the two proteins come to a close proximity (<10 Å), the split fragments of the fluorophore can reconstitute to its native state, consequently emitting a fluorescence signal (53). In the absence of an interaction between the two proteins, there is no reconstitution of the split fluorophores (53). Our group has successfully used BiFC previously to visualize complex formation between Nef and multiple host cellular proteins such as phosphofurin acidic cluster sorting protein-1, MHC-I, and SNX18 (54).

**Figure 3:**
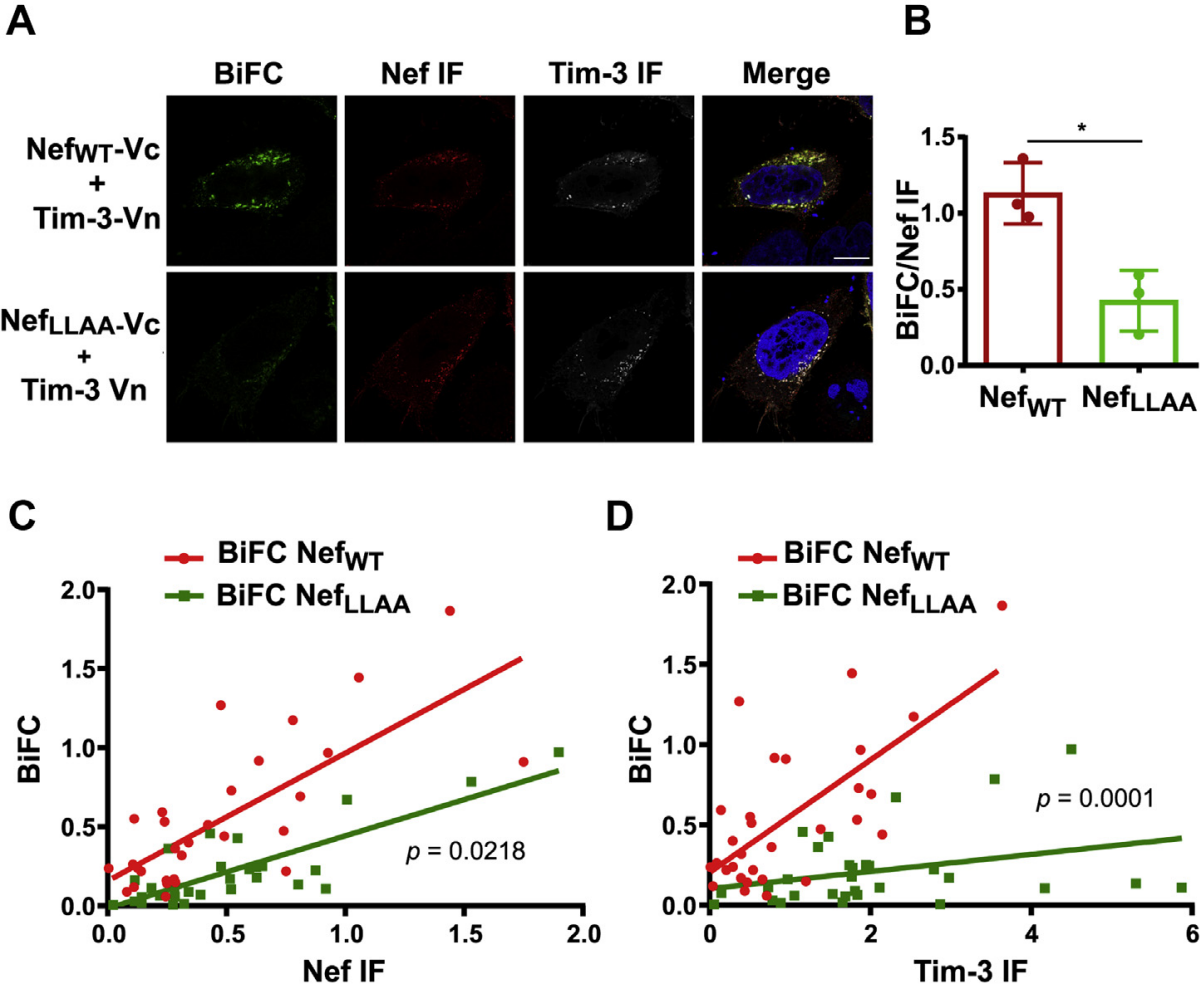
**Nef and Tim-3 are in close proximity within cells.***A*, HeLa CD4^+^ cells were transfected with Tim-3-FLAG-Vn (Tim-3-Vn) and Nef_WT_-Vc or Nef_LLAA_-Vc. Twenty-four hours later, cells were fixed, immunostained for FLAG (*white*) and HA (*red*), and mounted on DAPI-containing media for nuclear staining (*blue*), and BiFC (*green*) was observed using a Zeiss 880 Airyscan microscope. Images shown are representative cells from three independent experiments and 30 cells total. The scale bar is 10 μm. *B*, graph summarizing the BiFC signal intensity in HeLa CD4^+^ cells transfected with Tim-3-Vn and Nef_WT_-Vc or with Nef_LLAA_-Vc mutant. The values are normalized to Nef expression (HA staining). The *dots* represent three independent repeats each for a total of 30 cells. The bar graph shows the mean ± SD (∗*p* < 0.05). *C*, correlation of the BiFC signal intensity and Nef expression in HeLa CD4^+^ cells transfected with Tim-3-Vn and Nef_WT_-Vc or with Nef_LLAA_-Vc. The *p*-value indicates the significance for the difference in slope between Nef_WT_ and Nef_LLAA_. n = 3, 30 cells. *D*, correlation between BiFC and Tim-3 expression in HeLa CD4^+^ cells transfected with Tim-3-Vn and Nef_WT_-Vc or with Nef_LLAA_-Vc mutant. The *p*-value indicates the significance for the difference in the slope between Nef_WT_ and Nef_LLAA_, n = 3, 30 cells. BiFC, bimolecular fluorescence complementation; DAPI, 4′,6-diamidino-2-phenylindole; Nef_WT_-V_C_, Venus fluorophore was fused to Nef; Tim-3, T-cell immunoglobulin mucin domain-3; Tim-3-V_N_, FLAG-tagged Tim-3.

Accordingly, each half of a split Venus fluorophore was fused to Nef (Nef_WT_-V_C_) or FLAG-tagged Tim-3 (Tim-3-V_N_). Plasmids encoding the two split fluorophores were cotransfected into CD4^+^ HeLa cells, and intracellular FLAG and HA immunostaining was performed to stain for Tim-3- and Nef-expressing cells, respectively (Fig. 3*A*, panel 1). The coexpression of Nef_WT_-V_C_ and Tim-3-V_N_ resulted in reconstitution of the Venus fluorophore, demonstrating that Nef and Tim-3 are indeed in close proximity (Fig. 3*A*, green channel, panel 1). We also sought to determine whether the Nef_LLAA_ mutant is deficient in coming into close proximity with Tim-3, as it was unable to upregulate Tim-3 (Fig. 2). Accordingly, we cotransfected Tim-3-V_N_ and Nef_WT_-V_C_ or Nef_LLAA_-V_C_ into CD4^+^ HeLa cells, immunostained for FLAG (white) and HA (red), and looked for BiFC fluorescence (green) in dually transfected cells. We observed a marked decrease in BiFC fluorescence intensity in cells expressing Nef_LLAA_ compared with Nef_WT_ (Fig. 3*A*, green channel, panel 2). Furthermore, we quantified the BiFC fluorescence intensity and normalized it to the intensity of the Nef (HA) fluorescence for each condition and observed a statistically significant decrease in BiFC intensity of the Nef_LLAA_ mutant compared with Nef_WT_, suggesting that Nef_LLAA_ is indeed deficient in its ability to come in close proximity with Tim-3 in cells compared with Nef_WT_ (Fig. 3*B*).

Next, to validate the BiFC signal, we sought to determine whether an increase in Nef and Tim-3 immunofluorescence intensity positively correlated with an increase in BiFC intensity, as BiFC signal intensity is dependent on the expression level of the protein pair. Accordingly, we plotted the intensity of the green (BiFC), white (Tim-3), and red (Nef) fluorescence for cells expressing Tim-3 and Nef_WT_ or the Nef_LLAA_ mutant. As expected, an increase in the fluorescence intensities of Nef and Tim-3 correlated to the intensity of the BiFC signal, further validating the BiFC assay (Fig. 3, *C* and *D*). Furthermore, the rate of increase of the BiFC signal intensity was proportional to Nef and/or Tim-3 expression. This was significantly less for Nef_LLAA_ compared with Nef_WT_, providing further evidence that the dileucine motif Nef mutant is deficient in its ability to come into close proximity with Tim-3 (Fig. 3, *C* and *D*).

### The fate of upregulated Tim-3 in CD4^+^ T cells

**Figure 4:**
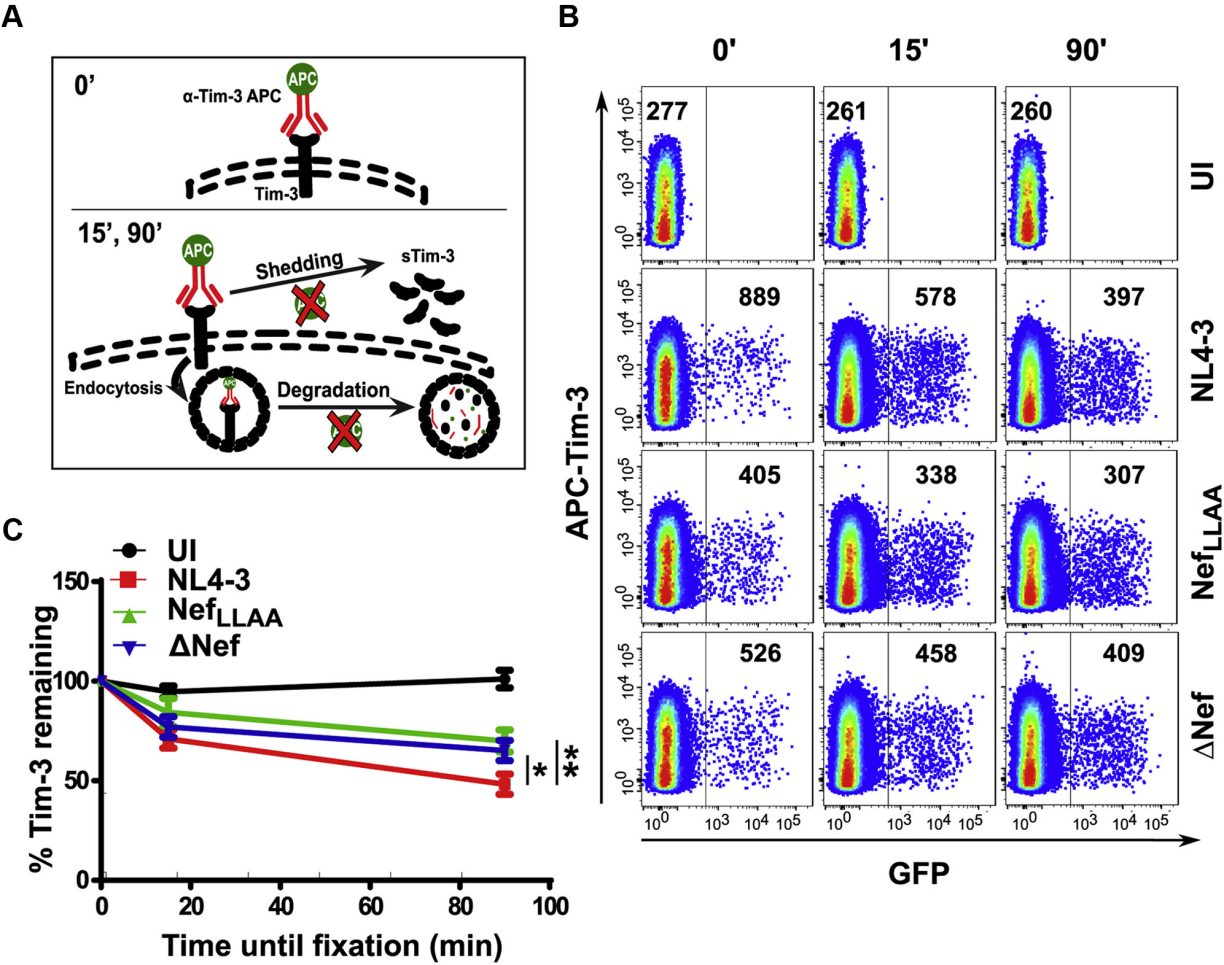
**Tim-3 receptor fate after Nef-mediated upregulation.***A*, schematic depicting the fate of Tim-3 (soluble Tim-3 [sTim-3]). *B*, purified CD4^+^ T cells from healthy donors were isolated using negative selection and infected with an eGFP-expressing NL4-3 or various Nef mutants (ΔNef or Nef_LLAA_). Forty-eight hours after infection, CD4^+^ T cells were stained at 4 °C using an anti-Tim-3 antibody and either fixed immediately or incubated at 37 °C for 15 or 90 min before fixation. The expression of labeled Tim-3 was analyzed using flow cytometry. Infected cells were gated based on eGFP expression. The mean fluorescence intensity of cell surface Tim-3 is indicated within each gate. *C*, graphical summary for the kinetics of Tim-3 receptor expression. The percentage of Tim-3 remaining on the cell surface was calculated by dividing the mean fluorescence intensity of the Tim-3 receptor at the 15- or the 90-min time point by the 0-min time point and multiplying the resulting value by 100. The 0-min time point was set to 100. ±SD of the mean is indicated (n ≥ 3 experiments; ∗*p* < 0.05, ∗∗*p* < 0.01). Tim-3, T-cell immunoglobulin mucin domain-3.

As we have established that Nef upregulates cell surface levels of Tim-3, we next sought to examine the fate of this upregulated Tim-3 after infection. As cell surface receptors can be degraded or shed into the extracellular milieu, we wanted to determine whether Nef affects the proportion of cell surface Tim-3 remaining intact (either on the surface or inside the cell) compared with cell surface Tim-3 that has been lost by shedding or degradation (Fig. 4*A*). Accordingly, we infected primary CD4^+^ T cells with constructs expressing Nef (NL4-3) or not (ΔNef) or a Nef_LLAA_ mutant and stained cell surface Tim-3 48 h after infection using an APC-conjugated Tim-3 antibody at 4 °C. Subsequently, cells were fixed immediately (0 min) and parallel wells of Tim-3-labeled cells were placed at 37 °C for 15 or 90 min, at which time, cells were fixed and analyzed for Tim-3 levels using flow cytometry (Fig. 4*A*). As expected, at the 0-min time point, infected CD4^+^ T cells had higher levels of Tim-3 compared with ΔNef virus or the Nef_LLAA_ mutant (Fig. 4*B*). Strikingly, at 15 min and 90 min, there was an ∼40% and ∼60% loss of original surface Tim-3 in cells infected with WT NL4-3, respectively (Fig. 4, *B* and *C*). In contrast, in CD4^+^ T cells infected ΔNef virus or the Nef_LLAA_ mutant, >70% of the original cell surface Tim-3 remained intact at the 90-min time point (Fig. 4, *B* and *C*). Therefore, although WT Nef upregulates cell surface levels of Tim-3, it also leads to an increased rate of loss of original cell surface Tim-3 than the Nef_LLAA_ mutant. As expected, there was no difference in Tim-3 levels in the uninfected controls at the different time points, indicating that cell surface Tim-3 is not readily lost in normal conditions (Fig. 4, *B* and *C*). Taken together, these data suggest that infection with a virus expressing WT Nef induces the loss of Tim-3 from the cell surface at a more rapid rate than ΔNef and Nef_LLAA._

### A broad-spectrum MMP inhibitor reduces Tim-3 shedding

**Figure 5:**
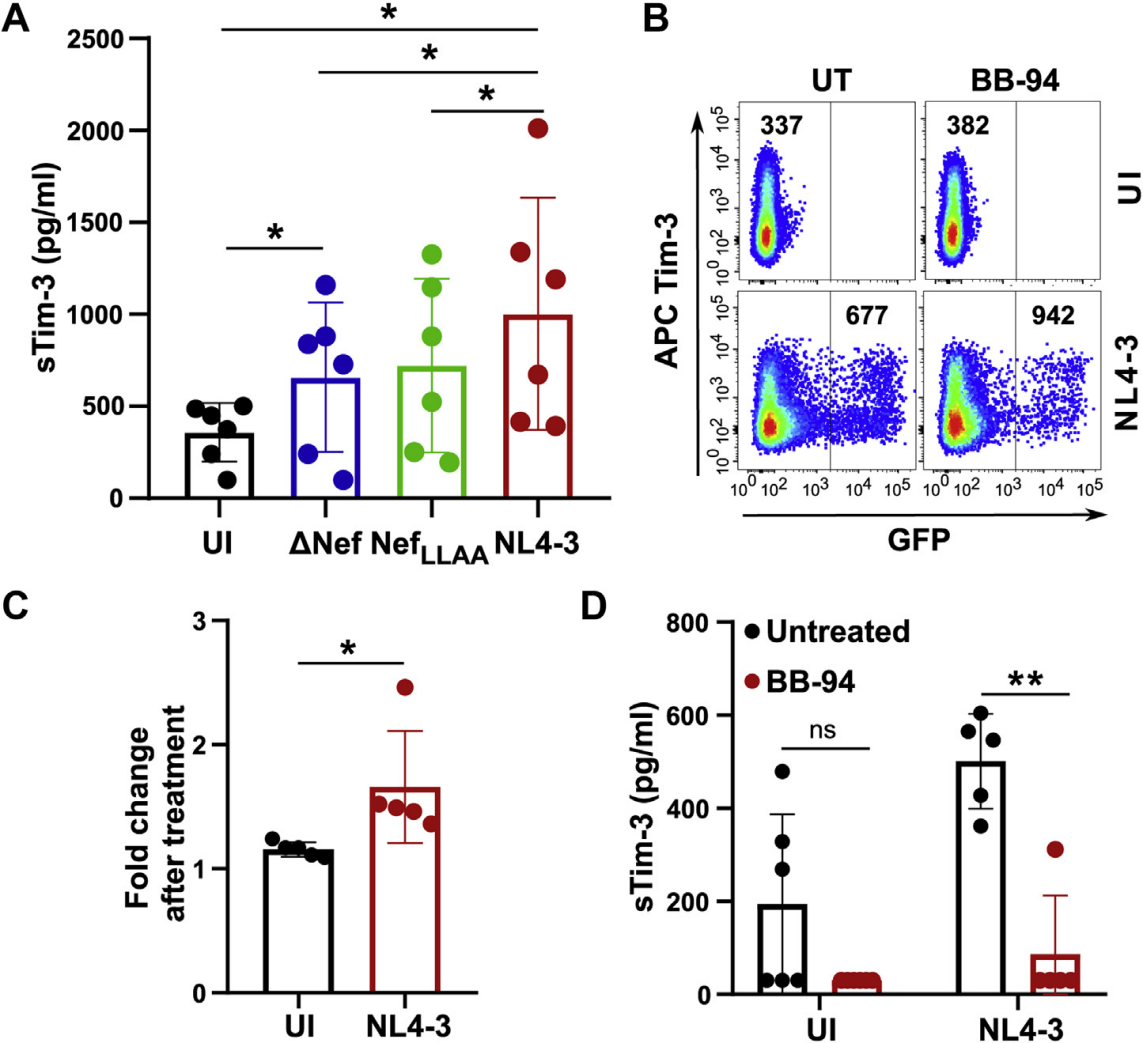
**Nef increases Tim-3 shedding from the cell surface.***A*, purified CD4^+^ T cells from healthy donors were isolated using negative selection and infected with an eGFP-expressing NL4-3 or the Nef mutants (ΔNef or Nef_LLAA_). Forty-eight hours after infection, cell culture supernatants were analyzed for soluble Tim-3 using a MAGPIX assay. Values are sTim-3 concentrations (pg/ml) ± SD of the mean (∗*p* < 0.05). *B*, purified CD4^+^ T cells from healthy donors were isolated using negative selection and infected with an eGFP-expressing NL4-3. Cells were left untreated or treated with 25 μM BB-94 soon after infection and cell surface Tim-3 analyzed 48 h after infection. The *number* indicates the mean fluorescence intensity of surface Tim-3. *C*, graph summarizing fold increase in Tim-3 cell surface levels after BB-94 treatment. The values were derived from the mean fluorescence intensity ratio between treated and untreated samples for the Tim-3 receptor. ±SD of the mean is indicated (n ≥ 3 experiments; ∗*p* < 0.05). *D*, purified CD4^+^ T cells from healthy donors were isolated using negative selection and infected with an eGFP-expressing NL4-3, and cells were left untreated or treated with 25 μM BB-94. Cell culture supernatants were analyzed for soluble Tim-3 using a MAGPIX assay. Values are sTim-3 concentrations (pg/ml) ± SD of the mean (n ≥ 3 experiments; ∗∗*p* < 0.01). BB-94, Batimastat; sTim-3, soluble Tim-3; Tim-3, T-cell immunoglobulin mucin domain-3.

A decrease in Tim-3 on the cell surface (Fig. 4) may be due to degradation or shedding of those molecules. Indeed, Tim-3 can be shed from the outer plasma membrane leaflet into a soluble form (55, 56, 57). ADAM10 and ADAM17, two major metalloproteinases involved in cleaving many receptors, act as the major Tim-3 sheddase by cleaving the ectodomain of Tim-3 (56, 58). Furthermore, levels of sTim-3 are significantly higher in the serum of individuals infected with chronic hepatitis B virus (59). Interestingly, the level of sTim-3 is also elevated in the plasma of chronic, untreated HIV-1-infected individuals and correlates positively with viral load and negatively with CD4^+^ T cell counts (56); however, viral factors triggering Tim-3 shedding have not been evaluated. Because Nef is known to enhance ADAM10 and ADAM17 activation and secretion, we next examined if Nef can induce Tim-3 shedding in NL4-3-infected CD4^+^ T cells [(60), Fig. 5*A*]. Accordingly, purified CD4^+^ T cells were infected with WT NL4-3 or constructs expressing Nef mutants (ΔNef and Nef_LLAA_). Subsequently, a magnetic bead–based Luminex assay was used to determine sTim-3 levels in cell culture supernatants. As expected, Tim-3 shedding was enhanced upon HIV-1 infection in comparison with uninfected controls (Fig. 5*A*). Furthermore, the levels of sTim-3 in the cell culture supernatant were significantly elevated in NL4-3-infected CD4^+^ T cells relative to the ΔNef virus or the Nef_LLAA_ mutant (Fig. 5*A*).

We next sought to understand the role of a broad-spectrum metalloproteinase inhibitor BB-94 in Tim-3 shedding. Accordingly, purified CD4^+^ T cells were infected with HIV-1 NL4-3 and either left untreated or treated with 25 μM BB-94. Forty-eight hours after infection, cell surface Tim-3 expression was measured using flow cytometry (Fig. 5*B*). Treatment with BB-94 significantly enhanced cell surface Tim-3 in NL4-3 infected CD4^+^ T cells (Fig. 5*C*). Because BB-94 treatment enhanced retention of cell surface Tim-3 in infected CD4^+^ T cells, we next tested if BB-94 treatment reduced Tim-3 shedding. Accordingly, purified CD4^+^ T cells were infected with NL4-3 virus and either left untreated or treated with 25 μM BB-94. Subsequently, sTim-3 was measured 48 h after treatment using a MAGPIX assay. Indeed, the level of sTim-3 was significantly reduced in NL4-3-infected CD4^+^ T cells upon treatment with BB-94, confirming Tim-3 retention on infected CD4^+^ T cells (Fig. 5*D*).

### The Tim-3-positive population is responsive to CD3/CD28 activation and robustly produces IFN-γ

**Figure 6:**
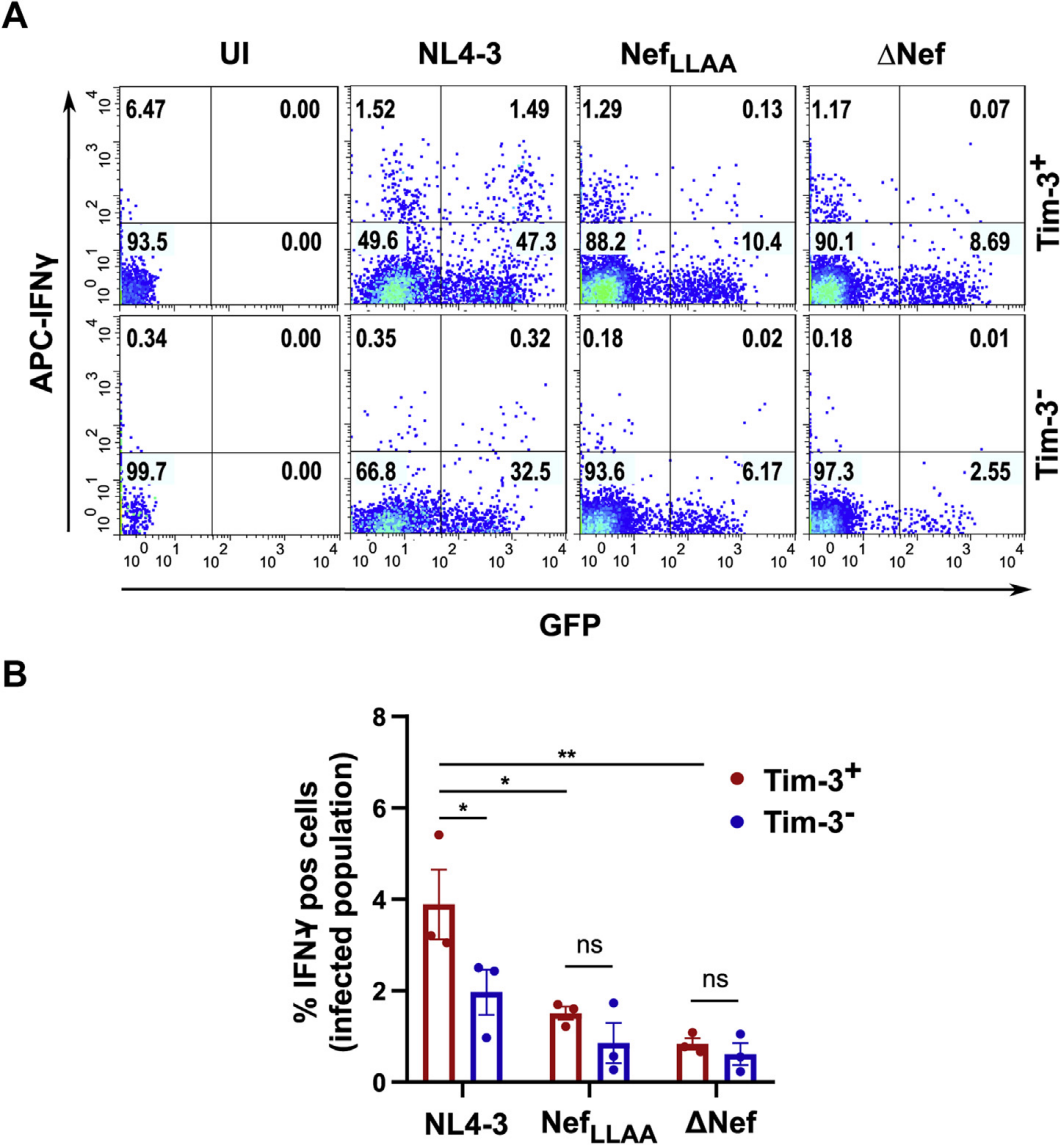
**Nef increases the frequency of IFN-γ-producing cells in the Tim-3**^**+**^**population.***A*, representative dot plots illustrating levels of IFN-γ in purified CD4^+^ T cells after T-cell receptor activation. PBMCs from healthy donors were revived without PHA/IL-2 activation, and CD4^+^ T cells were purified using negative selection and infected with an eGFP-expressing NL4-3 or the Nef mutants (ΔNef or Nef_LLAA_). Twenty-four hours after infection, cells were activated using anti-CD3/CD28 antibodies for 48 h. Cells were then incubated with Brefeldin A for 6 h and stained for intracellular IFN-γ before analysis by flow cytometry. *B*, graph summarizing the frequency of IFN-γ-producing cells in the infected Tim-3^+^ and Tim-3^−^ populations. ±SD of the mean is indicated (n = 3 experiments; ∗*p* < 0.05; ∗∗*p* < 0.01). IFN-γ, interferon-gamma; PBMCs, peripheral blood mononuclear cells; Tim-3, T-cell immunoglobulin mucin domain-3.

The expression of checkpoint receptors, such as Tim-3, on T cells impairs T cell functionality by reducing T cell proliferation, reducing effector function, and inhibiting cytokine release (3, 18, 19). Indeed, Tim-3-expressing CD8^+^ T cells from HIV-1-infected individuals display decreased responses to superantigens or to Gag peptides, indicating a state of exhaustion (11). However, although Tim-3 is best characterized as a negative regulator of T cell signaling, it can also enhance signaling through the T-cell receptor (TCR), generating optimal T cell activation (61, 62). Thus, we next assessed T cell functionality upon infection with HIV-1 constructs expressing various Nef proteins. We first performed a functional assay to determine the intracellular levels of IFN-γ in HIV-1-infected Tim-3^+^/CD4^+^ T cells. Accordingly, purified CD4^+^ T cells were infected for 24 h with NL4-3 virus or viruses expressing Nef variants (ΔNef and Nef_LLAA_), and TCR activation was induced by activating the CD3 and CD28-coupled pathways using monoclonal antibodies (Fig. 6*A*). The ability of the Tim-3^+^ and the Tim-3^−^ population to release IFN-γ was analyzed separately. Isotype control antibodies were used to gate the Tim-3-positive population from the Tim-3-negative population. Interestingly, upon infection with the NL4-3 WT virus, the Tim-3^+^ population intensely responded to TCR activation and actively produced IFN-γ when compared with the Tim-3^−^ population (Fig. 6, *A* and *B*). Moreover, Nef independently influenced T cell activation as a >2-fold increase in IFN-γ was observed in cells expressing WT Nef protein compared with cells infected with a Nef-deficient virus (ΔNef) or the Nef_LLAA_ mutant (Fig. 6, *A* and *B*). Overall, our results suggest that Nef-dependent Tim-3 upregulation on the surface of infected cells positively modulates the activation state of HIV-1-infected CD4^+^ T cells.

### The Nef proteins from T/F HIV-1 strains CH58 and CH77 upregulate Tim-3 on the surface of infected cells

**Figure 7:**
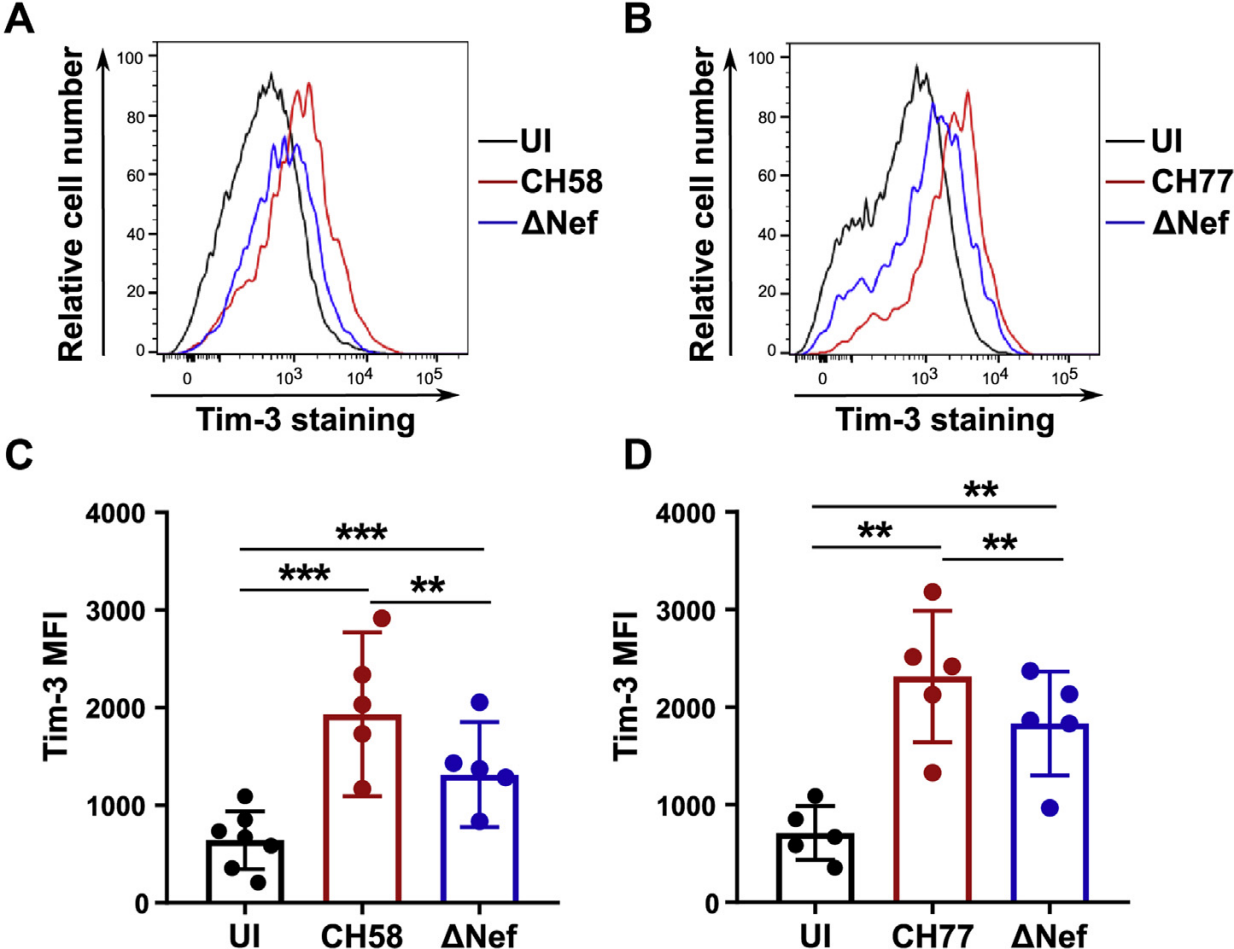
**The Nef protein from T/F HIV-1 strains modulate Tim-3 expression.***A* and *B*, purified CD4^+^ T cells from healthy donors were isolated using negative selection, activated using PHA/IL-2 stimulation, and infected with T/F viruses CH58 or CH77 and their ΔNef mutants. Forty-eight hours after infection, CD4^+^ T cells were stained using the Tim-3 antibody. The infected population was gated by intracellular p24 staining. *C* and *D*, the Tim-3 mean fluorescence intensity (MFI) in the uninfected (p24^−^) or the p24^+^ population is plotted graphically. These data were obtained in at least five independent experiments. (∗∗*p* < 0.01; ∗∗∗*p* < 0.001). T/F, transmitted/founder; Tim-3, T-cell immunoglobulin mucin domain-3.

We next sought to examine whether the Nef protein from T/F strains of HIV-1 also upregulate Tim-3 on the surface of infected cells. Accordingly, we infected primary CD4^+^ T cells with HIV-1 subtype B, T/F CH58 and CH77, either WT or defective for Nef expression (ΔNef). Subsequently, we examined cell surface levels of Tim-3 using flow cytometry (Fig. 7, *A* and *B*). Consistent with our observations using NL4-3 viruses, deleting Nef decreased cell surface levels of Tim-3, suggesting that Nef proteins from the T/F HIV-1 strains CH58 and CH77 significantly upregulates Tim-3 on the surface of infected primary CD4^+^ T cells (Fig. 7, *C* and *D*).

## Discussion

HIV-1 results in a chronic infection with persistent immune activation, leading to T cell exhaustion (63). Functionally, exhausted T cells lose their ability to proliferate, differentiate, and secrete proinflammatory cytokines (16). In addition, severely exhausted T cells are more likely to undergo apoptosis (16). Upon exhaustion, multiple cell surface receptors that modulate T cell signaling are upregulated (16). The HIV-1 accessory proteins Nef and Vpu have been shown to modulate the activity and localization of several host cell surface receptors, thereby regulating immune evasion and viral persistence (35, 64). In the present study, we defined the Nef-mediated cell surface modulation of checkpoint receptors in activated CD4^+^ T cells using HIV-1 NL4-3 and T/F strains CH58 and CH77. We demonstrate that Nef upregulates Tim-3 on the surface of infected CD4^+^ T cells and subsequently increases the shedding of Tim-3 to the extracellular milieu. These activities were primarily dependent on the previously characterized Nef dileucine motif (LL_164/165_), which is required for Nef’s interaction with the membrane trafficking coat protein complex AP-2. Nef hijacks host AP-2 to mediate other cell trafficking events such as downregulation of CD4 and SERINC5, key events implicated in antibody-dependent cellular cytotoxicity and infectivity, respectively. However, in both of these instances, Nef *downregulates* surface levels of CD4 and SERINC5 (51, 65). Conversely, our studies demonstrate a mechanism of Nef-dependent Tim-3 *upregulation*, akin to the previously reported Nef dileucine motif–dependent upregulation of the MHC class II–associated invariant chain (66). Although this mechanism has not yet been fully elucidated, it has been demonstrated that Nef decreases the rate of internalization of the MHC class II–associated invariant chain (66). Herein, we speculate that Nef expression is in fact failing to downregulate Tim-3 and instead increasing cell surface Tim-3 expression, which is subsequently shed. Although the Nef AP-2-binding site is implicated in this upregulation (Fig. 2), we speculate that alternative membrane trafficking regulators may be acting in concert or independent of AP-2 to increase cell surface Tim-3 expression. This hypothesis is consistent with the ability of the Nef dileucine motif to be in close proximity with Tim-3 in a process reinforcing the idea that the Nef–Tim-3 interaction modulates receptor localization. However, potential trafficking regulators involved in Tim-3 upregulation by Nef have yet to be determined.

We additionally observed that cell surface Tim-3 was lost upon infection with a virus expressing Nef more than after infection with a ΔNef or Nef_LLAA_ virus; however, this assay was unable to decipher whether this was due to degradation of Tim-3 or Tim-3 shedding. To further distinguish between these two possibilities, we used a MAGPIX assay to quantitate sTim-3 and demonstrate that Nef is a viral determinant driving Tim-3 shedding. This activity is consistent with that of the disintegrin-like metalloproteases (ADAMs), specifically the well-characterized ADAM10 and ADAM17 family members (58, 67). It has been previously shown that Tim-3 is a substrate for ADAM10 and ADAM17 (56). Furthermore, ADAM10 and ADAM17 activity and secretion have been shown to be modulated by Nef (60, 68, 69, 70). As such, we demonstrated that the broad-spectrum MMP inhibitor BB-94, which negatively affects Tim-3 shedding, blocks the release of sTim-3 in a Nef-dependent manner (71). A pronounced reduction in sTim-3 level was noticed upon treatment with the MMP inhibitor. Indeed, reduced Tim-3 shedding resulted in a consistent increase in the retention of membrane-bound Tim-3 on the cell surface. Thus, Nef and ADAM10 and ADAM17 both provide the framework to modulate the concentration of Tim-3 both on the cell surface and in the extracellular milieu. We demonstrate that Nef is a major factor involved in Tim-3 upregulation. However, our results suggest that Tim-3 upregulation upon infection is not entirely Nef-dependent. The Tim-3 fate experiment demonstrated a significant loss of labeled surface Tim-3 in cells infected with NL4-3 compared with the Nef_LLAA_ mutant. However, this effect was also observed in the Nef_LLAA_ mutant, albeit to a lesser extent. Intermediate phenotypes between uninfected and NL4-3-infected cells were also observed in the Tim-3 shedding assay. Although Tim-3 shedding was significantly higher in NL4-3 infected cells, we noticed a modest increase in shedding in cells infected with the ΔNef virus compared with the uninfected controls. This indicates that, although Nef is a major driver of Tim-3 modulation, there are also mechanisms independent of Nef that induce Tim-3 upregulation and shedding.

A hallmark of Nef-mediated regulation of cell surface proteins is its ability to form complexes with them (42, 72). In addition, we have previously shown using BiFC that another HIV-1 accessory protein, Vpu, is in close proximity with Tim-3 and mistrafficks it to the *trans*-Golgi network (32). It is therefore unsurprising that, in the present report, we demonstrate that Nef and Tim-3 are also in close proximity within cells. As the regulation of host cell surface proteins by HIV-1 Nef occurs primarily by forming complexes with them and inducing their mistrafficking within the cell, we speculate that the Nef-dependent upregulation of Tim-3 also involves mistrafficking of Tim-3 (34, 42, 64, 73).

Functionally, Tim-3 upregulation has been shown to dampen the immune response of CD4^+^ and CD8^+^ T cells by inducing anergy and cell death (18, 74). Specifically, Tim-3 can interact with Gal-9 *via* the N-terminal IgV domain, which can trigger apoptosis in T helper type 1 immune cells (20, 21, 27). A recent report demonstrated that blocking Tim-3 could enhance the response of cytotoxic T lymphocytes to purge the latent reservoir in Gal-9-treated CD4^+^ T cells, implying that neutralizing Tim-3 may be an effective approach to eliminate the virus reservoir (75). Further studies will define how Nef inhibitors can be used to block Tim-3 upregulation to potentially aid in elimination of the viral reservoir.

Although Tim-3 is commonly associated with T cell exhaustion, there is evidence that Tim-3 can have both stimulating and inhibitory effects on T cell signaling, and this distinction appears to be context-dependent. For example, the presence of a Tim-3 ligand such as CEACAM-1 induces the inhibitory effects of Tim-3; however, in the absence of ligand, Tim-3 enhances signaling through the TCR (61). Specific tyrosine residues within the cytoplasmic tail of Tim-3 are essential for regulating T cell activation and downstream signaling *via* the TCR (61, 76). Although HIV-1 preferentially infects activated CD4^+^ T cells, it remains unclear if HIV-1 is preferentially infecting CD4^+^ T cells expressing high levels of Tim-3 (77); however, we demonstrate that Nef can increase intracellular levels of IFN-γ in a Nef dileucine motif–dependent fashion. Because the dileucine motif is used for many Nef functions, we are unable to conclusively determine that this is Tim-3 dependent; however, this effect appears to be greater in Tim-3^+^ cells than Tim-3^−^ cells, suggesting Tim-3 upregulation by Nef activates infected CD4^+^ T cells. Overall, this is consistent with Nef’s previous role in T cell activation *via* nuclear factor kappa-light-chain-enhancer of activated b cells (78).

In addition to modulating T cell activation, Tim-family proteins have also been implicated in inhibiting viral release by binding PtdSer on virions using their IgV domains (31, 79). Interestingly, a recent report demonstrated a role of Nef in antagonizing Tim-family protein–mediated HIV-1 release by facilitating Tim-1 internalization from the plasma membrane (80). In addition, the report demonstrated that Nef antagonizes Tim-3-mediated inhibition of viral release in monocyte-derived macrophages (80). Further examination of the kinetics of Tim-3 on the cell membrane will be needed to assess the Nef-dependent role on Tim-3 localization in these cells.

We recently reported the role of Vpu on Tim-3 expression (32). Infection with two subtype B T/F molecular clones lacking Vpu resulted in higher surface levels of Tim-3 in CD4^+^ T cells (32). However, unlike the T/F molecular clones, in the present report, we show that NL4-3 Vpu was unable to modulate cell surface Tim-3 expression, suggesting differences in Vpu function between the lab-adapted strain NL4-3 and T/F strains. This is unsurprising, as previous studies have demonstrated that Vpu proteins from T/F viruses are 25% to 60% more effective in their function compared with NL4-3 Vpu (81). In addition, although some T/F viruses downregulate cell surface levels of HLA-C, NL4-3 does not (82).

The opposing effects played by HIV-1 Nef and Vpu in Tim-3 modulation indicate a complex functional interaction between the two accessory proteins on Tim-3. Together with previous reports from our group and others, we have identified that Nef upregulates Tim-3, whereas Vpu downregulates it. We hypothesize that this is due to the temporal regulation of these proteins—Nef is packaged in the virion and expressed early, whereas Vpu is expressed later in infection. Early in infection, it has been speculated that T cell activation is favored to facilitate viral replication and that this is partly influenced by Nef. However, later in infection, viral release would be prioritized, underscored by Vpu’s downregulation of tetherin. Furthermore, it has been shown that Nef and Vpu have contradictory effects on nuclear factor kappa-light-chain-enhancer of activated b cells, suggesting that these proteins may not have the same effect on T cell activation, possibly explaining their opposite effects on Tim-3 cell surface levels. Taken together, we speculate that Nef upregulates Tim-3 to further activate infected T cells, whereas Vpu downregulates Tim-3 to facilitate viral release. In summary, our study further describes the regulation of Tim-3 on the cell surface during HIV-1 infection by highlighting a key role for HIV-1 Nef in regulating surface levels of Tim-3.

## Experimental procedures

### PBMC isolation, CD4^+^ T cell purification, and cell culture

Human blood from healthy donors was collected in lithium heparin blood collection tubes (BD Biosciences). All human studies were approved by the University of Western Ontario and the CRCHUM Human Ethics Institutional Review Boards. All studies involving human subjects abide by the Declaration of Helsinki Principles. Peripheral blood mononuclear cells (PBMCs) were isolated using Ficoll density gradient separation (Sigma-Aldrich) and cryopreserved in 10% dimethyl sulfoxide (Sigma-Aldrich) and 90% fetal bovine serum (FBS; Wisent). Once thawed, PBMCs were cultured in complete RPMI 1640 media containing 10% FBS, 100 μg/ml penicillin-streptomycin, 1% sodium pyruvate, 1% nonessential amino acids, and 2 mM L-glutamine (HyClone). PBMCs were stimulated with 5 μg/ml phytohemagglutinin (PHA; Sigma-Aldrich) and 10 ng/ml of recombinant human interleukin-2 (IL-2; PeproTech) for 2 to 4 days before infection. CD4^+^ T cells were purified using the EasySep Human CD4^+^ T Cell Isolation Kit (#17952, Stemcell Technologies) according to the manufacturer’s protocol. The purity of CD4^+^ T cells was checked using flow cytometry and remained >90%.

HEK 293T (American Type Culture Collection) and CD4^+^ HeLa cells [NIH AIDS Reagent Program, Division of AIDS, NIAID, NIH, from Dr Richard Axel (83)] were grown in Dulbecco’s modified Eagle's medium containing 4 mM L-glutamine, 4500 mg/l glucose, and sodium pyruvate (DMEM, HyClone) and supplemented with 10% FBS (Wisent) and 1% penicillin and streptomycin (HyClone). SupT1 cells [NIH AIDS Reagent Program, Division of AIDS, NIAID, NIH, from Dr D. Ablashi] were grown in RPMI 1640 medium containing 100 μg/ml penicillin-streptomycin, 1% nonessential amino acids, and 2 mM L-glutamine and supplemented with 10% FBS (Wisent). Cells were grown at 37 °C in the presence of 5% CO_2_ and subcultured in accordance with supplier’s recommendations.

### DNA constructs

For pseudovirion production, the plasmids pCMV delta R8.2 encoding Gag/Pol and pMD2.G encoding vesicular stomatitis virus G (VSV-G) (both from Didier Trono, Addgene plasmids #12263 and #12259, respectively) were used in conjunction with previously described pNL4-3 ΔGag/Pol enhanced green fluorescent protein (eGFP) constructs (34, 41). The pNL4-3 virus and the corresponding isogenic variants were pseudotyped with VSV-G to achieve similar levels of infection.

Transmitted/founder (T/F) infectious molecular clones of patients CH58 and CH77 were inferred, constructed, and biologically characterized as previously described (84, 85, 86, 87, 88). Mutations were introduced using a QuikChange II XL site-directed mutagenesis kit (Agilent Technologies) as previously described (89, 90). Briefly, the Nef-defective CH58 and CH77 infectious molecular clone constructs were generated by introducing a premature stop codon at position 2 of its ORF. All mutations were confirmed by sequencing.

For microscopy, N-terminal DYKDDDK-tagged mouse Tim-3 (Tim-3-FLAG) was provided by Lawrence Kane (University of Pittsburgh, Pittsburgh, PA) and cloned into a pN1 backbone (Clontech) containing the VN-173 portion of the Venus fluorophore at the C terminus (91). HIV-1 NL4-3 Nef, as previously described (92, 93), was cloned into a pcDNA3.1(–) backbone (a kind gift from Thomas Smithgall, University of Pittsburgh, Pittsburgh, PA) and fused in-frame with a C-terminal hemagglutinin (HA) tag (YPYDVPDYA) and the VC-155 portion of the Venus fluorophore to generate Nef-HA-V_C_. To generate a mutant of the Nef dileucine motif, Nef-HA-V_C_ was mutated using Q5 site-directed mutagenesis (New England Biolabs), replacing LL_164–165_ with AA_164–165_ to generate Nef_LLAA_-HA-V_C_. All cloning and mutations were confirmed by sequencing (London Regional Genomics Centre).

### Pseudovirus production and infection

Pseudovirions were produced in HEK 293T cells by transfection using PolyJet (FroggaBio) with pCMV delta R8.2, pMD2.G, and pNL4-3 ΔGag/Pol eGFP constructs, as described previously (34). Pseudovirus harvesting was done 48 h after transfection by centrifuging the cell culture supernatants for 15 min at 1500 rpm and subsequent filtration using a 0.45-μm filter. The filtered supernatant was supplemented with an additional 10% FBS before storage at −80 °C. PBMCs or purified CD4^+^ T cells were spinoculated at 3000 rpm for 2 h at room temperature (RT) with pseudoviruses in the presence of 8 μg/ml polybrene.

### Surface marker staining for flow cytometry

HIV-1-infected PBMCs or purified CD4^+^ T cells were stained with fluorescently labeled anti-human antibodies (1:50 dilution) in the cell stain buffer (BioLegend) for 30 min at 4 °C, washed twice using the cell stain buffer, fixed in 2% paraformaldehyde (PFA), washed again once in PBS, and re-suspended in PBS before flow cytometry. Cell staining was quantified with a BD FACSCanto II cytometer. The data were analyzed using FlowJo software (version 10.7.1, Treestar). The following antibodies (BioLegend) were used for staining surface receptors: APC PD-1 (#329907), APC TIGIT (#372705), Alexa Fluor 647 LAG-3 (#369303), APC Tim-3 (#345012), APC-Cy7 Tim-3 (#345026), APC CD4 (#317416), and APC/Cy7 HLA-A, B, C (#311426). The following isotype controls were used in this study: APC Mouse IgGκ, Alexa Fluor 647 Mouse IgG1κ, APC Mouse IgG2aκ, APC Mouse IgG2bκ, APC/Cy7 Mouse IgG1κ, and APC/Cy7 Mouse IgG2aκ.

### Intracellular IFN-γ staining for flow cytometry

For intracellular staining of IFN-γ, PBMCs were revived in RPMI for 6 h without PHA/IL-2, and CD4^+^ T cells were purified and infected. Twenty-four hours after infection, the T cell receptor was activated using an immobilized anti-CD3 antibody (10 μg/ml, clone OKT3, Ultra-LEAF purified, #317326, BioLegend) and soluble anti-CD28 antibody (5 μg/ml, clone CD28.2, Ultra-LEAF purified, #302934, BioLegend). Brefeldin A–treated cells were surface stained for Tim-3, fixed, and permeabilized using the Cytofix/Cytoperm solution (#554722, BD Biosciences) 48 h after activation. Intracellular staining was performed using manufacturers' staining protocol (BD Biosciences) using the APC IFN-γ antibody (#502511, BioLegend). Cells were washed twice using perm/wash buffer before analysis by flow cytometry. The data were analyzed using FlowJo software (version 7.6, Treestar).

### Determination of Tim-3 fate

HIV-1-infected purified CD4^+^ T cells were washed twice in the cell stain buffer and stained with the APC-conjugated anti-Tim-3 antibody (1:50 dilution) for 30 min in the cell stain buffer at 4 °C. Cells were then washed twice using the cell stain buffer and either fixed immediately (0 min) in 2% PFA or shifted to 37 °C for different periods of time (15 or 90 min) in complete RPMI media and fixed using 2% PFA before analysis by flow cytometry. The data were analyzed using FlowJo software (version 10.7.1, Treestar). The percentage of Tim-3 remaining on the cell surface was analyzed by dividing the mean fluorescence intensity (MFI) at the 15- or 90-min time point by the 0-min time point and multiplying the resulting value by 100.

### T/F HIV-1 flow cytometry

Primary human CD4^+^ T cells were isolated, activated, and cultured as previously described (94, 95). Briefly, PBMCs were obtained by Ficoll density gradient centrifugation from whole-blood samples obtained from healthy donors. CD4^+^ T lymphocytes were purified from resting PBMCs by negative selection using immunomagnetic beads according to the manufacturer’s instructions (Stemcell Technologies). CD4^+^ T cells were activated with PHA (10 μg/ml) for 48 h and maintained in RPMI 1640 complete medium supplemented with recombinant IL-2 (100 U/ml).

To achieve similar levels of infection among all viruses, VSV-G-pseudotyped HIV-1 viruses were produced in HEK 293T cells and titrated as previously described (96). Viruses were then used to achieve a level of infection of ∼10% of the total primary CD4^+^ T cells at 48 h after infection. PHA/IL-2-activated primary CD4^+^ T cells from healthy HIV-1-negative donors were spinoculated at 800*g* for 1 h in 96-well plates at 25 °C.

The following antibodies were used as primary antibodies for cell surface staining of primary CD4^+^ T cells: allophycocyanin (APC)-anti-human CD366 (Tim-3) (clone F38-2E2; BioLegend), APC-mouse IgG2 (clone MOPC-173; BioLegend) was used as matched IgG isotype controls. Cell surface staining was performed as previously described (95, 96). Binding of antibodies to cell surface Tim-3 (7.5 μg/ml) was performed at 48 h after infection. Infected cells were stained intracellularly for HIV-1 p24 using the Cytofix/Cytoperm fixation/permeabilization kit (BD Biosciences) and a fluorescent anti-p24 mAb (PE-conjugated anti-p24, clone KC57; Beckman Coulter/Immunotech). The percentage of infected cells (p24^+^) was determined by gating the living cell population using Aqua Vivid viability dye staining (Thermo Fisher Scientific). Samples were acquired on an LSR II cytometer (BD Biosciences), and data analysis was performed using FlowJo v10.7.1 (Treestar).

### Microscopy

CD4^+^ HeLa cells (5 × 10^5^ cells) were seeded onto sterile glass coverslips. Twenty-four hours later, cells were transfected with the appropriate plasmid using PolyJet transfection reagent (FroggaBio). Twenty-four hours after transfection, the BiFC fluorophore was matured by incubating the cells at RT for 1 h. The cells were then washed three times with 1× PBS, fixed in 4% PFA for 15 min at RT, and washed again three times in 1× PBS. Immunostaining was performed as described previously (92). Cells were permeabilized in 1× PBS containing 0.02% Triton X for 10 min at RT, washed three times in 1× PBS, and incubated with a drop of Image-iT FX Signal Enhancer (Invitrogen) for 30 min at RT. Coverslips were washed three times in 1× PBS and incubated in 5% BSA (Tocris Bioscience) in PBS containing 0.01% Triton X-100 (blocking buffer) for 15 min and then incubated with primary rat anti-FLAG antibody (1:400; clone L5; BioLegend) and mouse anti-HA antibody (1:200; clone 16B12; BioLegend) for 1 h 10 min. Coverslips were washed three times in the blocking buffer for 5 min each and subsequently incubated in donkey anti-rat Alexa Fluor 647 and donkey anti-mouse Alexa Fluor Cy3 (1:500 and 1:400, respectively; Jackson ImmunoResearch) for 1 h 10 min. Antibodies were diluted in the blocking buffer. Coverslips were washed three times with 1x PBS for 5 min each before being mounted on slides with Fluoromount-G containing 4′,6-diamidino-2-phenylindole (SouthernBiotech) and imaged on a Zeiss LSM 880 microscope at 63× magnification (numerical aperture 1.4) using the fluorescein (FITC), cyanine 3 (Cy3), cyanine 5 (Cy5), and 4′,6-diamidino-2-phenylindole filter settings on the fast Airyscan mode. Images were subsequently processed using Zeiss Airyscan Processing (2D, auto) on the ZEN Blue software. Quantification of MFI was done using Fiji/ImageJ by subtracting MFI of each channel of a cell in the nontransfected condition (as a background value) from the MFI determined by ImageJ for the various conditions.

### Batimastat treatment

Purified CD4^+^ T cells were infected with eGFP-expressing pseudoviruses and treated with 25 μM Batimastat (BB-94) (Abcam) or with dimethyl sulfoxide as vehicle. At 48 h after infection, cell culture supernatants were collected to determine soluble Tim-3 (sTim-3) levels using MAGPIX multiplex assay (see below). Simultaneously, cells were processed to determine cell surface Tim-3 expression using flow cytometry. The data were analyzed using FlowJo software (version 10.7.1, Treestar). The fold increase in Tim-3 cell surface levels was calculated by taking the Tim-3 MFI ratio between treated and untreated samples.

### Multiplex assay

A Tim-3 Human ProcartaPlex Simplex Kit (Thermo Fisher, cat# EPX01A-12219-901) was used to measure sTim-3 concentrations according to manufacturer’s instructions. Tim-3 levels from PBMC supernatants infected with HIV NL4-3 or Nef variants were measured in duplicate. Plates were washed by adding 100 μl of the wash buffer (0.05% Tween 20, 20 mM Tris HCl, pH 7–8, in 1× PBS). After washing and incubation, the wells were resuspended with 50 μl of the assay buffer and read by the MAGPIX multiplex assay hardware (Luminex, S/N 14283705). The MAGPIX program was created and run using xPONENT V4.2 (Luminex). The output was recorded using Bio-Plex Manager v6.1 software (Bio-Rad).

### Statistics

All statistical analyses were performed using GraphPad Prism 8.0.2 (GraphPad Software, Inc). Two-way ANOVA was used for multiple comparisons, and two-tailed Student’s *t* test was used for two-group comparisons. For microscopy analysis, to determine whether the slopes of the correlations were significantly different, a linear regression test was used. The following *p* values were considered significant: ∗*p* < 0.05, ∗∗*p* < 0.01, ∗∗∗*p* < 0.001, and ∗∗∗∗*p* < 0.0001. Data variance is represented as ±SD of the mean.

## Data availability

All data are contained within the article.

## Supporting information

This article contains supporting information.

## Conflict of interest

The authors declare that they have no conflicts of interest with the contents of this article.
