# Supplementary material for: The HIV-1 accessory protein Nef increases surface expression of the checkpoint receptor Tim-3 in infected CD4+ T cells
Source: J Biol Chem. 2021 Aug 4;297(3):101042. doi: 10.1016/j.jbc.2021.101042 (PMC8390549; doi:10.1016/j.jbc.2021.101042)
Supplement: Supplemental Figure S1 [file mmc1.pdf]

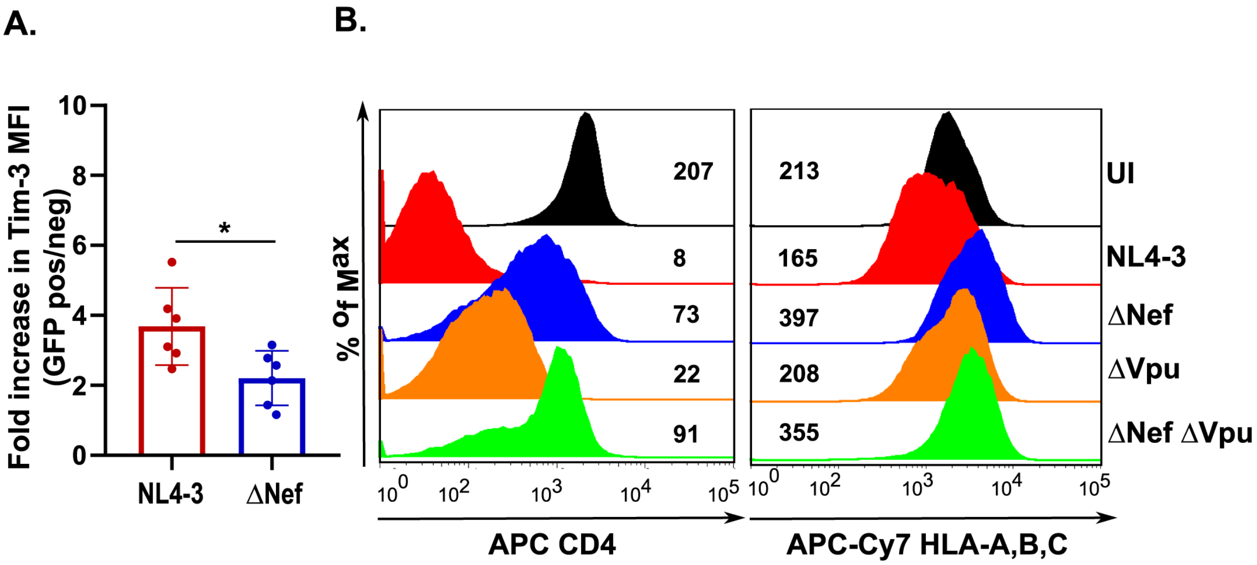

Suppl. Figure 1

Suppl figure 1: A) **Graph summarizing the fold increase in Tim-3 levels**. The values were derived by taking a ratio of the mean fluorescence intensity of surface Tim-3 in the infected (GFP-positive) and uninfected (GFP-negative) population upon infection with NL4-3 or the isogenic virus lacking Nef expression. ± standard deviation of the mean is indicated (n ≥ 5 experiments from ≥3 donors; *, $p<0.05$). B) **Flow cytometric analysis of CD4 and MHC-I cell surface expression after HIV-1 infection.** Representative histograms illustrating Nef-mediated CD4 and MHC-I cell surface downregulation. SupT1 cells were infected either with an eGFP expressing NL4-3 or a mutant lacking the expression of Nef (ΔNef) or Vpu (ΔVpu) or both (ΔNef ΔVpu). Forty-eight h post infection, cells were surface stained for CD4 and MHC-I and analyzed using flow cytometry. Infected cells were gated based on eGFP expression and the mean fluorescence intensities are indicated.
